# Supplementary material for: Real-World Outcomes of Chemoradiotherapy in Patients with Stage II/III Non-Small-Cell Lung Cancer in the Durvalumab Era: An Observational Study
Source: Cancers (Basel). 2025 Jul 29;17(15):2498. doi: 10.3390/cancers17152498 (PMC12345666; doi:10.3390/cancers17152498)
Supplement: Supplementary file 1 [file cancers-17-02498-s001.zip › cancers-3747453-supplementary.pdf]

Supplement

Characteristic	Overall N = 72	CRT with durvalumab N = 35	CRT without durvalumab N = 37
**Age at diagnosis** (years) [1]	66 (46, 83)	66 (46, 83)	67 (50, 82)
**Sex**			
male	54 (75%)	26 (74%)	28 (76%)
female	18 (25%)	9 (26%)	9 (24%)
**Smoking status** (pack years) [1]	34 (0, 60)	35 (0, 60)	33 (0, 60)
**CCI group** [2]	2.00 (0.00, 8.00)	2.00 (0.00, 8.00)	1.00 (0.00, 7.00)
**Karnofsky Index** (%)[3]	80 (50, 100)	80 (70, 100)	80 (50, 100)
**PD-L1 status** (%) [3]	5 (1, 60)	30 (5, 75)	1 (1, 2)
**PD-L1 status +/- durvalumab**			
PD-L1 <1% + durvalumab	1 (1.4%)	1 (2.9%)	0 (0%)
PD-L1 <1% + no durvalumab	19 (26%)	0 (0%)	19 (51%)
PD-L1 ≥1% + durvalumab	34 (47%)	34 (97%)	0 (0%)
PD-L1 ≥1% + no durvalumab	10 (14%)	0 (0%)	10 (27%)

Characteristic	Overall<br>N = 72	CRT with durvalumab<br>N = 35	CRT without durvalumab<br>N = 37
Unknown	8 (11%)	0 (0%)	8 (22%)
**T-stage**			
1	2 (2.9%)	2 (5.9%)	0 (0%)
2	8 (11%)	3 (8.8%)	5 (14%)
3	19 (27%)	7 (21%)	12 (33%)
4	41 (59%)	22 (65%)	19 (53%)
**N-stage**			
0	14 (20%)	7 (20%)	7 (19%)
1	12 (17%)	6 (17%)	6 (17%)
2	27 (38%)	13 (37%)	14 (39%)
3	18 (25%)	9 (26%)	9 (25%)
**UICC-stage**			
IIb	4 (5.6%)	1 (2.9%)	3 (8.1%)
IIIa	27 (38%)	12 (34%)	15 (41%)
IIIb	27 (38%)	15 (43%)	12 (32%)

Characteristic	**Overall** N = 72	**CRT with durvalumab** N = 35	**CRT without durvalumab** N = 37
IIIc	14 (19%)	7 (20%)	7 (19%)
**Histology**			
adenocarcinoma	20 (29%)	8 (23%)	12 (34%)
large cell carcinoma	1 (1.4%)	1 (2.9%)	0 (0%)
neuroendocrine carcinoma	1 (1.4%)	0 (0%)	1 (2.9%)
squamous cell carcinoma	48 (69%)	26 (74%)	22 (63%)
**Chemotherapy**			
Carboplatin monotherapy	4 (5.6%)	3 (8.6%)	1 (2.7%)
Carboplatin/ Paclitaxel	25 (35%)	10 (29%)	15 (41%)
Cisplatin/ Vinorelbine	43 (60%)	22 (63%)	21 (57%)
**Total radiation dose** (Gy) [4]	66.00, 65.17 (60.00, 66.00)	66.00, 65.00 (60.00, 66.00)	66.00, 65.33 (60.00, 66.00)

[1] Mean (Min, Max); n (%);[2] Median (Min, Max);[3] Median (Q1, Q3);[4] Median, Mean (Min, Max)

Supplement Table S1. Complete baseline characteristics of patients receiving chemoradiotherapy with and without durvalumab.

Table Adverse Events

Characteristic	N = 80[1]
Dysphagia	36 (47%)

Characteristic	N = 80[1]
Coughing	23 (30%)
Dyspnea	18 (24%)
Erythema	17 (22%)
Nausea/ Vomiting	13 (17%)

[1] n (%)

Stratified by durvalumab treatment

Characteristic	CRT+durvalumab N = 35[1]	CRT-durvamluab N = 37[1]
Dysphagia	18 (51%)	16 (47%)
Coughing	11 (31%)	12 (35%)
Dyspnea	7 (20%)	10 (29%)
Erythema	9 (26%)	8 (24%)
Nausea/ Vomiting	4 (11%)	7 (21%)

[1] n (%)

Supplement Table S2. Frequency of Selected Adverse Events in the Study Cohort.

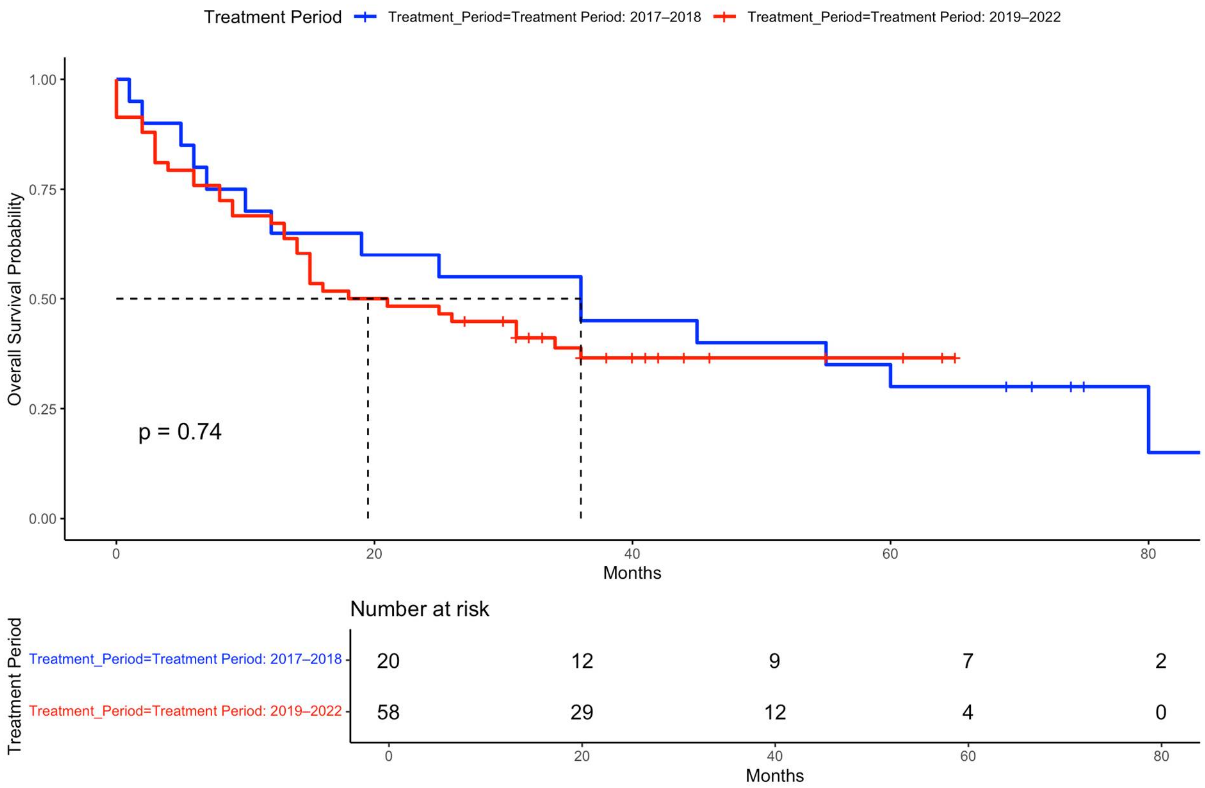

Figure S1. Kaplan-Meier curve for overall survival (OS) stratified by treatment period.

	PFS (adjusted)			OS (adjusted)		
**Characteristic**	**HR**	**95% CI**	**p-value**	**HR**	**95% CI**	**p-value**
**CCI-group**			0.032			0.009
>2	—	—		—	—	
<=2	0.50	0.26, 0.96		0.40	0.20, 0.82	
**Karnofsky Index**			<0.001			0.003
≤80	—	—		—	—	
>80	0.29	0.14, 0.62		0.31	0.13, 0.70	
**durvalumab**			0.010			0.004

	PFS (adjusted)			OS (adjusted)		
**Characteristic**	**HR**	**95% CI**	**p-value**	**HR**	**95% CI**	**p-value**
yes	—	—		—	—	
no	3.75	1.45, 9.73		4.60	1.74, 12.2	
**PD-L1 status**			0.043			0.024
<1	—	—		—	—	
≥1	2.31	0.83, 6.38		5.08	1.64, 15.8	
Unknown	0.53	0.16, 1.75		2.00	0.52, 7.76	
**Treatment period**			0.3			0.076
2017-2018	—	—		—	—	
2019-2022	1.56	0.68, 3.59		2.25	0.89, 5.68	
**N-Status**						0.065
0				—	—	
1				1.25	0.34, 4.54	
2				3.25	1.15, 9.17	
3				1.56	0.52, 4.71	

Abbreviations: CI = Confidence Interval, HR = Hazard Ratio

Table S3. Multivariable Cox regression models for PFS and overall survival OS adjusted for treatment period. Hazard ratios (HR), 95% confidence intervals (CI), and p-values are reported for

each clinical characteristic. Both models were selected using stepwise variable selection based on Akaike's Information Criterion (AIC), with the treatment period (2017–2018 vs. 2019–2022) forcibly included to account for evolving clinical practice and durvalumab availability. CCI = Charlson Comorbidity Index.
